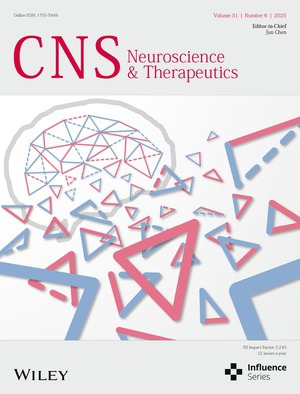# Front Cover

**DOI:** 10.1111/cns.70474

**Published:** 2025-06-02

## Abstract

The cover image is based on the article *Network Topography Alterations in Alzheimer's Disease: Insights From Motif Changes via Multisite Datasets (N = 3262)* by Hongwei Li et al., https://doi.org/10.1111/cns.70428.